# Over-Expression of Either MECP2_e1 or MECP2_e2 in Neuronally Differentiated Cells Results in Different Patterns of Gene Expression

**DOI:** 10.1371/journal.pone.0091742

**Published:** 2014-04-03

**Authors:** Marija Orlic-Milacic, Liana Kaufman, Anna Mikhailov, Aaron Y. L. Cheung, Huda Mahmood, James Ellis, Peter J. Gianakopoulos, Berge A. Minassian, John B. Vincent

**Affiliations:** 1 Molecular Neuropsychiatry & Development Lab, Campbell Family Mental Health Research Institute, The Centre for Addiction & Mental Health, Toronto, Ontario, Canada; 2 Developmental & Stem Cell Biology Program, The Hospital for Sick Children, Toronto, Ontario, Canada; 3 Program in Genetics & Genomic Biology, The Hospital for Sick Children, Toronto, Ontario, Canada; 4 Division of Neurology, Department of Paediatrics, The Hospital for Sick Children, Toronto, Ontario, Canada; 5 Department of Psychiatry, University of Toronto, Toronto, Ontario, Canada; 6 Institute of Medical Science, University of Toronto, Toronto, Ontario, Canada; King Faisal Specialist Hospital and Research center, Saudi Arabia

## Abstract

Mutations in *MECP2* are responsible for the majority of Rett syndrome cases. MECP2 is a regulator of transcription, and has two isoforms, MECP2_e1 and MECP2_e2. There is accumulating evidence that MECP2_e1 is the etiologically relevant variant for Rett. In this study we aim to detect genes that are differentially transcribed in neuronal cells over-expressing either of these two MECP2 isoforms. The human neuroblastoma cell line SK-N-SH was stably infected by lentiviral vectors over-expressing MECP2_e1, MECP2_e2, or eGFP, and were then differentiated into neurons. The same lentiviral constructs were also used to infect mouse Mecp2 knockout (Mecp2^tm1.1Bird^) fibroblasts. RNA from these cells was used for microarray gene expression analysis. For the human neuronal cells, ∼800 genes showed >three-fold change in expression level with the MECP2_e1 construct, and ∼230 with MECP2_e2 (unpaired t-test, uncorrected p value <0.05). We used quantitative RT-PCR to verify microarray results for 41 of these genes. We found significant up-regulation of several genes resulting from over-expression of MECP2_e1 including *SRPX2*, *NAV3*, *NPY1R*, *SYN3*, and *SEMA3D*. *DOCK8* was shown via microarray and qRT-PCR to be upregulated in both SK-N-SH cells and mouse fibroblasts. Both isoforms up-regulated *GABRA2*, *KCNA1*, *FOXG1* and *FOXP2*. Down-regulation of expression in the presence of MECP2_e1 was seen with *UNC5C* and *RPH3A*. Understanding the biology of these differentially transcribed genes and their role in neurodevelopment may help us to understand the relative functions of the two MECP2 isoforms, and ultimately develop a better understanding of RTT etiology and determine the clinical relevance of isoform-specific mutations.

## Introduction

Rett syndrome (RTT; MIM#312750) is a progressive neurodevelopmental disorder that affects females almost exclusively, with a frequency of ∼1∶10000 births. The overwhelming majority of Rett syndrome cases are caused by mutations in the Methyl-CpG-binding protein 2 gene, *MECP2* [1]. The MECP2 protein has two isoforms, MECP2_e1 and MECP2_e2, which differ in exon usage and amino terminal amino acid sequences [2], [3]. There is much evidence to suggest that MECP2_e1 may be the etiologically relevant isoform in Rett Syndrome based on its expression profile in the brain and because, to-date, no mutations in Rett individuals have been discovered that affect MECP2_e2 exclusively, while mutations have been found in exon 1 that exclusively impact on MECP2_e1 [4], [5]. Overall, this would suggest that MECP2_e1 may be more relevant to Rett syndrome.

The two MECP2 isoforms exhibit different regional and temporal expression patterns in mouse brain [6], with MECP2_e1 more abundant throughout the brain, and MECP2_e2 less abundant and more restricted. It is frequently assumed that there are no functional differences between the two isoforms, although the evidence to support this assumption is limited. A recent study using complementation with isoform-specific *MECP2* transgenes in *MECP2*-null mice suggests that both isoforms can prevent development of the Rett-like phenotype in the mice, and thus may substitute for each other, and are likely to perform similar cellular functions [7]. *In silico* analysis predicts that MECP2_e1 is a more stable (and N-acetylated) protein and has a longer half-life (220 hrs) than MECP2_e2 (unacetylated; 65 hrs; [8]). However, the addition of the N-terminal eGFP tag to the MECP2_e2 constructs by Kerr et al. [7] may lead to N-acetylation (MECP2_e1 transgene was tagged at C-terminus), and double the predicted half-life of the protein. Also, the degree of phenotypic rescue for the two isoforms in the Kerr et al study was not fully concordant, particularly for motor phenotypes, with the MECP2_e1 transgene more effective. Thus, while very similar, with >95% sequence identity, there still may be important functional differences between the two isoforms.

Also, in contrast to the Kerr et al study, recent studies of isoform-specific MECP2 knockouts in mice appear to show that knockout of MECP2_e2 has no detrimental effect on normal neurodevelopment and behavior [9], whereas knockdown of MECP2_e1 fully recapitulates the neurological symptoms seen in Rett syndrome [10]. Thus, there is now convincing data to support the hypothesis that disruption of MECP2_e1 rather than MECP2_e2 leads to RTT.

We hypothesize that the two isoforms regulate the transcription of different sets of genes, and for MECP2_e1 these will be enriched for genes relevant to the neurodevelopmental phenotype. To investigate this hypothesis we have infected human SK-N-SH cell lines with lentiviral constructs encoding either MECP2_e1 or MECP2_e2 or a control construct, and differentiated stably infected cells into neurons [11]. One of the major advantages of this approach is that we are able to look selectively at neuronal response, rather than at a heterogeneous group of cells including glia and astrocytes, which may greatly dilute the response from neurons. We have used gene expression microarrays supported with quantitative real time RT-PCR to catalogue genes that are either up or down-regulated under the effects of over-expression of either isoform in the differentiated cells. In addition, in order to investigate the effect of over-expression of MECP2_e1 or MECP2_e2 in MECP2 null background, we used these lentiviral vectors to stably infect primary mouse fibroblasts derived from male MECP2 knockout mice. Although human neuronal cells with no functional MECP2 expressed would have been desirable, such cells were not available for the study. As our experiments studied the effects of over-expression of MECP2, the results are also relevant to MECP2 duplication syndrome (MIM #300260).

## Materials and Methods

### Research Ethics Statement

Human brain tissue was obtained and used following informed written consent from next of kin and approval from the Research Ethics Board at the Centre for Addiction and Mental Health (http://www.camh.ca/en/research/research_ethics/research_ethics_board_general_information/Pages/reb_general_information.aspx).

### Cell Lines

Human neuroblastoma cell line SK-N-SH (ATCC, Manassas, VA, USA) was grown in Eagle’s Minimal Essential Medium (Invitrogen, Carlsbad, CA, USA) supplemented with 10% FBS (Wisent, St. Bruno, QC, Canada) and Penicillin-Streptomycin (Invitrogen, Carlsbad, CA, USA) Primary mouse fibroblasts isolated from MECP2 knockout mice Mecp2^tm1.1Bird^ (Jackson laboratories, ME, USA) were generated in the Ellis lab by standard procedures, and were grown in Minimal Essential Medium (Invitrogen, Carlsbad, CA, USA) supplemented with 20% FBS and Penicillin-Streptomycin. Cells were kept at 37°C in humid atmosphere with 5% CO_2_.

### Infections

The recombinant lentiviruses that we used to infect subconfluent SK-N-SH cells were described previously by Rastegar et al. [12]. These are self-inactivating lentiviruses encoding either MECP2_e1, MECP2_e2 or eGFP under the control of the EF1α promoter. The optimal multiplicity of infection (MOI) for infection of SK-N-SH cells was determined by infecting SK-N-SH cells and primary mouse MECP2 knockout fibroblasts by the eGFP-encoding lentiviral vector. The MOI of 20 resulted in over 90% of infected SK-N-SH cells, while the optimal MOI for primary mouse fibroblasts was determined to be 100. The optimal MOI for each cell line was used for infection of these cell lines with all lentivirus constructs. The infections were performed in the presence of 100 μg/ml polybrene (Sigma-Aldrich). Three independent infections with each lentiviral construct were performed for each cell line, providing three biological replicates for each treatment. Three days after infection the virus-containing medium was removed and replaced with fresh growth medium.

### Neuronal Differentiation

Neuronal differentiation of infected SK-N-SH cells was achieved by treatment of cells with retinoic acid (Cedarlane Laboratories, Burlington, ON, Canada) and herbimycin A (Cedarlane Laboratories, Burlington, ON, Canada), using a modification of the protocol previously described [11]. Retinoic acid was added to the medium at 10 μM concentration seven days post-infection, and cells were grown in the presence of retinoic acid for 96 h, with medium being replaced with fresh retinoic-acid containing medium every two days. Retinoic acid treatment increases the number of neuronally committed cells in the SK-N-SH culture (Preis et al. 1988). After 96 hours, herbimycin A was added to the growth medium at 236 nM concentration, and cells were grown in the presence of both herbimycin A and retinoic acid for 72 hours, with medium being replaced with fresh medium after 48 hours. Herbimycin A was used to clear the undifferentiated cells from the culture [11].

### RNA Isolation and Reverse Transcription

RNA was isolated from stably infected neuronally differentiated cells and from stably infected primary mouse fibroblasts using TRIzol (Invitrogen, Carlsbad, CA, USA), in accordance with the manufacturer’s protocol. The concentration of total RNA was determined by NanoDrop spectrophotometry. Isolated RNA was stored at −80°C. The reverse transcription was performed using 2 μg of total RNA, SuperScript III reverse transcriptase (Invitrogen, Carlsbad, CA, USA) and random primers (Invitrogen, Carlsbad, CA, USA) in accordance with the manufacturer’s instructions.

### Microarray Gene Expression Analysis

RNA isolated from stably infected neuronally differentiated SK-N-SH cells and primary mouse fibroblasts was used for microarray gene expression analysis on human and mouse Agilent arrays, respectively (Agilent Technologies, Santa Clara, CA, USA). For SK-N-SH cells, the Agilent Whole Human Genome Oligo44K (#14850) two-color microarrays were used, and for mouse fibroblasts the Agilent Whole Mouse Gene Expression 44K (#14868) two-color arrays were used. Microarray hybridizations, data collection, and analysis of results were performed as a service by the University Health Network (UHN) Microarray Centre (http://www.microarrays.ca/). Each hybridization was performed in triplicate. Therefore, there were three technical replicates for each biological replicate. Hybridizations were two-color, and human and mouse reference RNAs (Agilent, Santa Clara, CA, USA) were used as controls for SK-N-SH and mouse primary fibroblasts, respectively. Fold changes in gene expression were calculated relative to eGFP-lentivirus-treated cells. Both ANOVA analysis and unpaired t-tests were used for statistical analysis. BioVenn (http://www.cmbi.ru.nl/cdd/biovenn/index.php [13]) was used to identify the overlap in differentially expressed genes between different treatments. We used DAVID Gene Ontology (http://david.abcc.ncifcrf.gov/ [14], [15]) for functional clustering of differentially expressed genes.

Microarray data from this study has been deposited in the NCBI GEO, and has been assigned accession numbers GSE54258 (human/SK-N-SH) and GSE54261 (mouse/ko fibroblast).

### Real-Time RT-PCR

Real-time RT-PCR was performed on ABI 7500 or Viia7™ machines (Life Technologies, Foster City, CA, USA). Expression of *NAV3*, *FOXP2* and *PTCHD1* was examined using TaqMan Gene Expression Assays (Hs00372108_m1, Hs00362817_m1, and Hs00288486_m1, respectively) and TaqMan PCR MasterMix (Life Technologies, Foster City, CA, USA). Primers with probes for analysis of MECP2_e1 and MECP2_e2 expression were custom ordered from IDT and their sequences are provided in Table 1. Expression of other genes of interest was validated by using custom-made primers and SYBRgreen PCR master mix (Life Technologies, Foster City, CA, USA). Primer sequences are also provided in Table 1. Two housekeeping genes, *HPRT* and *GAPDH*, were initially used as endogenous controls, but after it was established that the expression of these two genes did not vary between samples, only one endogenous control gene, *HPRT*, was used. TaqMan Gene Expression Assays for *HPRT* and *GAPDH* (Hs99999909_m1 and Hs02758991_g1, respectively) were used to quantify *HPRT* and *GAPDH* transcripts used as endogenous control for relative quantification of *NAV3*, *FOXP2* and *PTCHD1* expression. TaqMan Gene Expression Assay for *Tbp* (Mm00446973_m1) was used as an endogenous control to determine expression changes in MECP2_e1 and MECP2_e2 after lentiviral infection. Sequences of *HPRT* primers used with SYBRgreen chemistry are provided in Table 1. Each real-time PCR reaction was done in triplicate. Real-time RT-PCR results were analyzed using ABI 7500 Software v2.0.1 or ViiA7 RUO software. Microsoft Excel was used for statistical analysis of real-time RT-PCR data by F-test and to plot the data. RNA isolated from an adult human brain frontal cortex sample, kindly provided by Dr. Stephen Kish (following approval from the Research Ethics Board at the Centre for Addiction and Mental Health (http://www.camh.ca/en/research/research_ethics/research_ethics_board_general_information/Pages/reb_general_information.aspx), and written consent from next of kin) was used as a positive control for expression of genes of interest in the brain.

**Table 1:** Primers used for real-time RT-PCR and end-point PCR.

Gene	Forward Primer	Reverse Primer
ELAVL3	ggctacgggtttgtgaactatt	atggatgctgaactgggtct
SPRX2	ctttctgctgttcttcctaactcc	tgggacctcctctgcatatact
NDN	atgtggtacgtgctggtcaa	acttcttgtagctgccgatga
UNC5C	agtgaatgggttcatcagaagg	caccagtaatcttcaggtccaaa
RPH3A	atgaggacatgcaaaggaaga	tttcttgagggagaatctggtc
ITGA3	gtcatcaacatcgtccacaaga	gtaagcaaagcacagctccact
GABRB1	gcttctctctttccctgtgatg	tttgagcaatctgtccactgtc
CNTN4	gggaatgtaaagcaaatggaag	tgttgagtgttccttgctcaat
DLGAP2	gtgcaagtggaagatgagaagc	tttgtcctccgtggtgatgt
NPY1R	ggatctgagcaggagaaatacc	tgaactgaacaatcctctttgg
SYN3	tcgatgatgcccatacagac	gtcacataggcagctaggttca
CHL1	ttcattcttaccgggttgtc	tgattgttggaacctgttga
GABRA2	aattctgcttgccgtttcagag	atcttcttggatgttagccagcac
KCNA1	ctccaccaaagccaagataaac	caaagtgctgttaccgacagag
PTPRZ1	ttgcattcagctcctctgtg	ataggaccagccaatctcttca
BMP7	agcctgcaagatagccatttc	ggaaagatcaaaccggaactct
GRIN2A	ttatctcctcccacaccttcgt	tgcatgatcttcagcatgacc
FMN2	cagggaaccgtgtaatcagaa	caatgagtgtgtgggttgactaa
REST	cgagtatcactggaggaaacattta	atatgggcgttctcctgtatga
NR0B1	gtgctttctttccaaatgctg	ccactggagtccctgaatgta
NEFM	acgtcaagatggctctggata	agtgatgcttcctgcaaatgt
GRM8	ttggctgcaagttaggatcac	aattggacctttccttcctgtt
LGICZ	ccatccctcttcaacgtcaa	tgtgtatcgcaggatgtcca
FOXG1	caacggcatctacgagttcat	agcacttgttgagggacagatt
SEMA3D	gaccatgttgtttcttccagtca	cagtccttctgatgaacccaaa
DOCK8	cacgagaagtatatgtccctcaca	tattgtaatgttccgggctga
mDock8	tgttctctgtcacctacccatct	ttactctttccaccatcgcttt
HPRT	tggtcaggcagtataatccaaa	tcaagggcatatcctacaacaa
mHprt	ttcctcatggactgattatggac	gtaatccagcaggtcagcaaa
BMX	agatgtgtggagaaagtaaatctcg	ctgacttcggctctcttcattt
mBmx	tgcagtaaccaactggaaagaa	atattcccagcaaaccagtcat
RNF2	ctcagtcacagcattgaggaag	gtcaccattatcttctgctccac
mRnf2	gggtcttagcaaggatcaacaa	ttgcctcgctgtaatctgttc
LPHN3	caactatggcaggactgatga	actgcacactgggttctgttat
mLphn3	cagatggagaacattcggtgt	ggacatgggtctggaaatacat
GPR85	aggaatatcagactgcgaatcacc	ttgccatcagaatatgcgaaga
mGpr85	tggtggatttcaggtttcgta	cttccgtttgatttgccatc
SMCR8	agcttattggcttgcagagagtg	tttgttgtcaaggtccaggatg
mSmcr8	cggtgaagcactggatttct	tgcttgtataacggctgtaacg
COQ10B	atacggccttgagaagggtagt	attgaggtatgtagtggaagagttctg
mCoq10b	ctaccggcttcacaaagatga	ttcttgcattgagtacctgacg
TMC4	ttcacagaagtcacccagacaga	tccgtcttagttccagagccata
mTmc4	tctacttgctgtgcatcctacg	aagctgagaacactcggtgact
ITGBL1	cacaagacatcatctgctctaatg	gcattctctatcgtcacactcac
mItgbl1	gtgagtgccatgagtggatatg	acttggttgggtactgacaagc
ZBTB10	ggacccgcaactacaagaaa	cctctgatggtatttctgactcct
mZbtb10	ggacccgcaactacaagaaa	cctctgatggtatttctgactcct
MECP2_e1	tgcttgccctctttctcttc	ggcgaggaggagagactg
MECP2_e1 probe: 56-FAM/tccagggcc/ZEN/tca		
MECP2_e2	tgcttgccctctttctcttc	tggtagctgggatgttaggg
MECP2_e2 probe: 56-FAM/tcctggtct/ZEN/tctg		

Primers specific for mouse cDNAs are indicated by the prefix “m”.

### Microscopy

To determine the percentage of eGFP positive cells after infection, sterile glass cover slips coated with Poly-D-Lysine (Sigma-Aldrich) were put in tissue culture flasks prior to cell plating and infection. Seven days after infection, the medium was removed, and cells were washed with PBS and fixed with 4% PFA. Glass cover slips were recovered, placed on microscope slides and, after cell nuclei were counterstained with DAPI, examined for percentage of eGFP positive cells by fluorescence microscope (Eclipse E600, Nikon) in the green fluorescent field.

To examine the percentage of cells with neuronal morphology during the course of retinoic acid and herbimycinA treatment, SK-N-SH cells were grown and treated with differentiation medium in microscope slide chambers. When the treatment procedure was completed, cells were fixed with methanol, stained with Coomassie blue, and examined in the bright light field using Nikon microscope (Eclipse E600, Nikon).

## Results

### Stably Infected Cells Over-Express MECP2 Isoforms

Cell lines of neuronal origin, like SK-N-SH cells, as well as primary cell cultures are known to be refractory to commonly used transfection reagents. In addition, our experiment required a longer time frame than the one provided by transient transfection. Therefore, we used recombinant lentiviruses to achieve stable over-expression of human MECP2 isoforms e1 or e2, or stable expression of control gene eGFP in human neuroblastoma cell line SK-N-SH and primary mouse MECP2 knockout fibroblasts. The empirically determined optimal MOI for these cells resulted in >90% of infected cells, based on eGFP expression (Figure S1).

Stably infected SK-N-SH cells were differentiated into neurons by retinoic acid and herbimycinA treatment [11]. After being grown in differentiation medium for seven days, as described in Materials and Methods, more than 90% of cells in culture exhibited neuronal morphology (Figure S2).

Infected cells were harvested and RNA was isolated from primary mouse MECP2 knockout fibroblasts seven days after infection, and from SK-N-SH cells after the differentiation treatment was completed. Expression of MECP2 isoforms in primary mouse MECP2 knockout fibroblasts and overexpression of MECP2 isoforms in neuronally differentiated SK-N-SH cells was confirmed by real-time RT-PCR.

**Figure 1:**
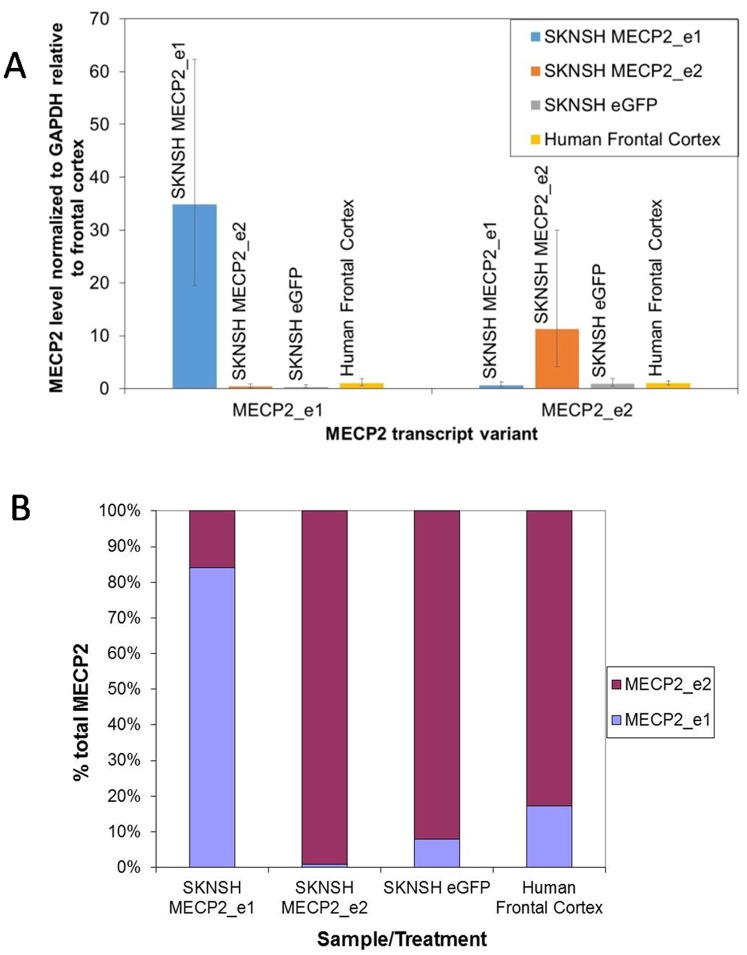
Expression of *MECP2* isoforms in SK-N-SH cells stably infected with recombinant lentiviruses. A) Infection with the MECP2_e1 vector increases MECP2_e1 mRNA level ∼35-fold, compared with the brain frontal cortex and ∼100-fold compared with the eGFP control infected cells. Infection with the MECP2_e2 vector increases *MECP2_e2* mRNA level 11-fold compared with the brain frontal cortex and the eGFP control. Relative quantities of MECP2_e1 and MECP2_e2 transcripts for each treatment/sample are indicated in data labels, below sample names; B) In cells infected with the MECP2_e1 vector, *MECP2_e1* transcript is ∼5 times more abundant than the *MECP2_e2* transcript. In cells infected with the MECP2_e2 vector, the *MECP2_e2* transcript is ∼100 times more abundant than the *MECP2_e1* transcript. In cells infected with the eGFP control and in the brain frontal cortex, the *MECP2_e2* transcript is ∼9 times more abundant than the *MECP2_e1* transcript.

Neuronally differentiated SK-N-SH cells infected with the control eGFP lentiviral vector express both transcript variants of MECP2. The level of MECP2_e1 is approximately 3-fold lower than in the frontal cortex of the human brain, while MECP2_e2 levels are similar between brain and neuronally differentiated neuroblastoma cells. Stable infection with MECP2-encoding recombinant lentiviruses results in overexpression of MECP2_e1 or MECP2_e2. In SK-N-SH cells infected with the MECP2_e1 vector, MECP2_e1 transcript is 35 times more abundant than in the brain frontal cortex (∼100-fold increase relative to SK-N-SH cells infected with the control eGFP-expressing lentiviral vector), and 5 times more abundant than the MECP2_e2 transcript. In SK-N-SH cells infected with the MECP2_e2 vector, MECP2_e2 transcript is 11 times more abundant than in the brain frontal cortex and SK-N-SH cells infected with the eGFP control vector, and approximately 100 times more abundant than the MECP2_e1 transcript (Figure 1A and 1B).

Infection of MECP2 knockout primary mouse fibroblasts at MOI = 100 results in ∼80% of infected cells (Figure S1). MECP2 lentivirus vectors induce expression of MECP2 isoforms in mouse MECP2 knockout fibroblasts (Figure S3).

### Predominant Expression of MECP2_e1 or MECP2_e2 Results in Distinct Gene Expression Profiles

An uncorrected analysis using one way ANOVA (p<0.01) identified 1098 genes differentially expressed between SK-N-SH (neuronally differentiated) infected with MECP2_e1, MECP2_e2 or eGFP lentiviral vectors (Table S1) and 489 genes differentially expressed between mouse Mecp2 knockout fibroblasts infected with MECP2_e1, MECP2_e2 or eGFP lentiviruses (Table S2). However, correction for multiple testing using the Benjamini-Hochberg false discovery rate (FDR) test showed no significant differences (p<0.05). Using a t-test (p<0.05) ∼800 genes were identified with at least three-fold change in expression level (up- or down-regulation) when MECP2_e1 was over-expressed in SK-N-SH cells, in comparison with the eGFP control. When MECP2_e2 was over-expressed in SK-N-SH cells, levels of ∼230 genes changed at least 3-fold. Correction for FDR, however, showed no significant differences. For microarray t-test analysis results, please refer to Table S3 and Table S4.

Six genes are at least two-fold up-regulated by MECP2_e1 in both SK-N-SH cells and primary mouse MECP2-null fibroblasts: *TSHR*, *BMX*, *DOCK8*, *COQ10B*, *RNF2* and *LPHN3*. *TMC4* is ∼2-fold up-regulated by MECP2_e2 expression in both SK-N-SH cells and primary mouse fibroblasts. Four genes are at least two-fold down-regulated by MECP2_e1 in both SK-N-SH cells and primary mouse fibroblasts: *ITGBL1*, *GPR85*, *KCNJ9* and *ZBTB10*. *SMCR8* is ∼three-fold downregulated by MECP2_e2 in both SK-N-SH cells and primary mouse fibroblasts.

#### Gene ontology analysis of SK-N-SH expression data

**Figure 2:**
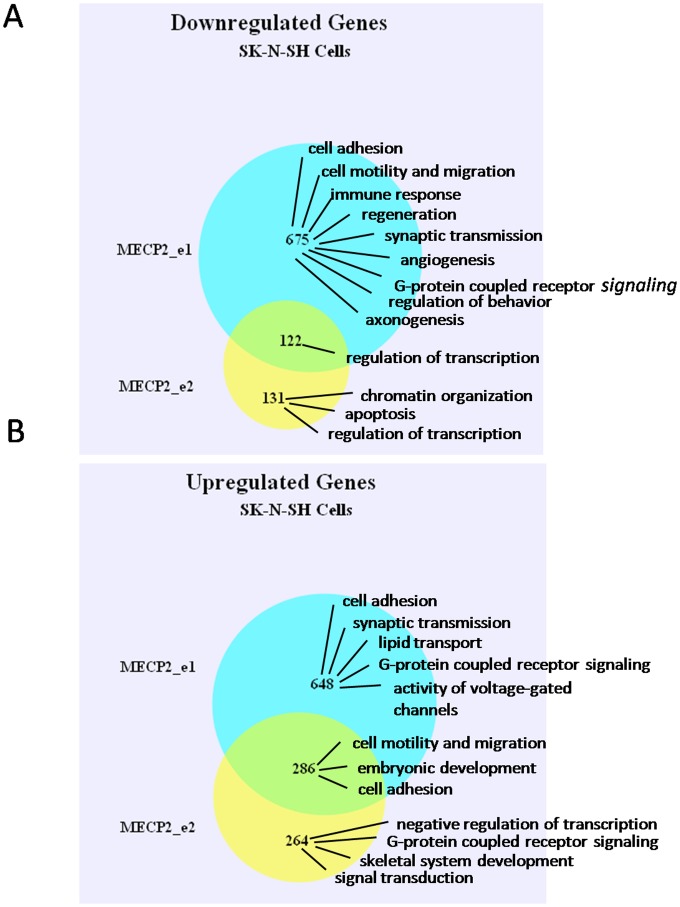
Venn diagrams showing the number of genes identified by microarray analysis as down-regulated (A) or up-regulated (B) at least two fold by overexpression of MECP2_e1 isoform (blue), MECP2_e2 isoform (yellow), or both isoforms (intercept) in SK-N-SH cells. Biological processes that indicated gene sets are enriched for, as identified by DAVID Functional Clustering, are indicated on the right hand side of Venn diagram.

For the purpose of gene ontology analysis using DAVID, we used a cut-off of two-fold change and p<0.05. Over-expression of MECP2_e1 in neuronally differentiated SK-N-SH cells resulted in at least two-fold down-regulation of 797 genes, while over-expression of MECP2_e2 resulted in at least two-fold down-regulation of 253 genes. Of these, 122 genes were downregulated by both MECP2_e1 and MECP2_e2, and according to DAVID functional clustering, these genes are mainly involved in transcriptional control (see Table S5 and SKNSH S1). The 675 genes that were down-regulated only by MECP2_e1 are involved in cell adhesion, cell migration and motility, synaptic transmission, axonogenesis, G-protein coupled receptor signaling, regulation of behavior, immune response, regeneration, and angiogenesis (Table S5 and SKNSH S2). The 131 genes that were down-regulated only by MECP2_e2 function in chromatin organization, regulation of transcription and apoptosis (Figure 2A; Tables S5 and SKNSH S3). Over-expression of MECP2_e1 in neuronally differentiated SK-N-SH cells up-regulated, at least two fold, 934 genes, while over-expression of MECP2_e2 up-regulated, at least two-fold, 550 genes. Of these, 286 genes are up-regulated by both MECP2_e1 and MECP2_e2 and are involved in cell adhesion, cell motility, cell migration, and embryonic development (Tables S5 and SKNSH S4). The 648 genes up-regulated at least two-fold only by MECP2_e1 were significantly enriched for synaptic transmission, activity of voltage-gated channels, G-protein coupled receptor signaling, cell adhesion, and lipid transport (Tables S5 and SKNSH S5). The 264 genes up-regulated at least two-fold by MECP2_e2 alone were significantly enriched for negative regulation of transcription, G-protein coupled receptor signaling, signal transduction, and skeletal system development (Figure 2B; Table S5 and file SKNSH S6).

#### Gene ontology analysis of mouse knockout fibroblast expression data

**Figure 3:**
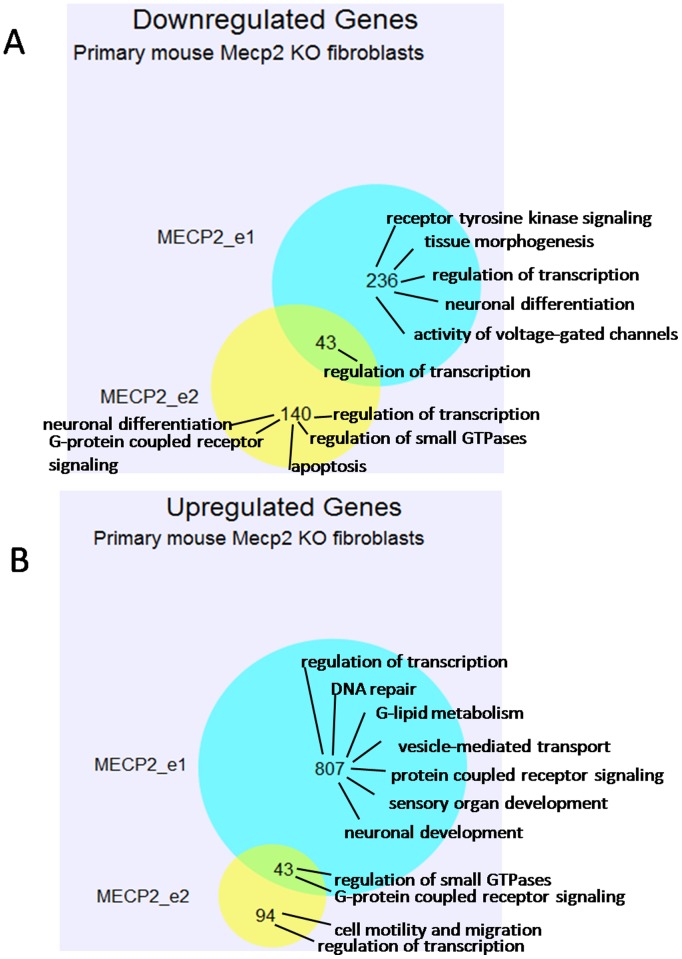
Venn diagram showing the number of genes identified by microarray analysis as down-regulated (A) or up-regulated (B) at least two fold by overexpression of MECP2_e1 isoform (blue), MECP2_e2 isoform (yellow), or both isoforms (intercept) in primary fibroblasts derived from Mecp2 knockout mice. Biological processes that indicated gene sets are enriched for, as identified by DAVID Gene Ontology Functional Clustering, are indicated on the right hand side of Venn diagrams.

Using the uncorrected t-test data (p<0.05), as FDR correction showed no significant results, the expression of MECP2_e1 in primary mouse MECP2 knockout fibroblasts resulted in >two-fold down-regulation of 236 genes (for microarray t-test results, please refer to Table S6 and Table S7). By DAVID functional clustering, these genes are mainly involved in the regulation of signaling by receptor tyrosine kinases, tissue morphogenesis, regulation of transcription, neuronal differentiation, and regulation of activity of voltage gated channels (Table S8 and Mouse S1). Expression of MECP2_e2 in primary mouse MECP2 knockout fibroblasts resulted in >two-fold down-regulation of 140 genes, mainly involved in regulation of transcription, neuronal differentiation, G-protein coupled receptor signaling, regulation of small GTPases and apoptosis (Table S8 and Mouse S2). Forty three genes are>two-fold down-regulated by both MECP2_e1 and MECP2_e2 expression and are mainly involved in transcriptional regulation (Figure 3A; Table S8 and Mouse S3). At least two-fold up-regulation of 807 genes was detected when MECP2_e1 was expressed in primary mouse MECP2 knockout fibroblasts. These genes are involved in transcriptional regulation, neuronal development, sensory organ development, DNA repair, lipid metabolism, G-protein coupled receptor signaling and vesicle-mediate transport (Table S8 and Mouse S4). Expression of MECP2_e2 in primary mouse MECP2 knockout fibroblasts resulted in >2-fold up-regulation of 94 genes involved in transcriptional regulation and cell motility and migration (Table S8 and Mouse S5). Forty three genes are at least two-fold up-regulated by both MECP2_e1 and MECP2_e2 expression and are mainly implicated in G-protein coupled receptor signaling and regulation of small GTPases (Figure 3B; Table S8 and Mouse S6).

### Quantitative Validation of Microarray Results by RT-PCR

**Figure 4:**
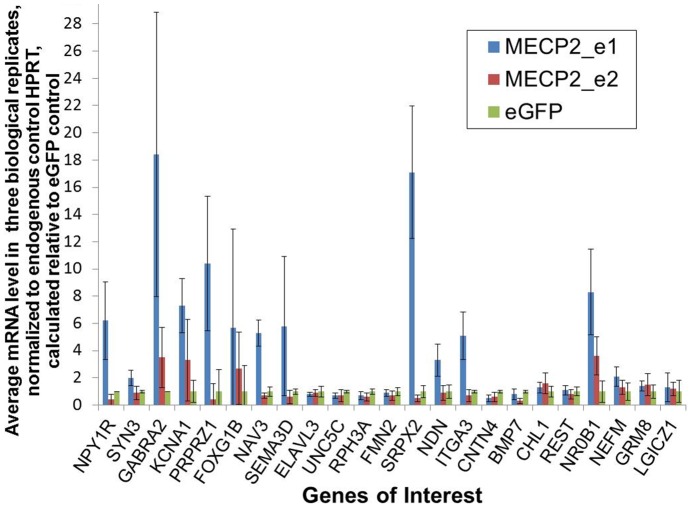
Real-time RT-PCR-based quantification of expression of 23 genes, identified to be differentially regulated by microarray analysis, in SK-N-SH cells stably infected with either MECP2_e1, MECP2_e2 or eGFP-expressing lentiviral vectors. Expression of target genes was normalized to HPRT endogenous control and calculated relative to eGFP control treatment.

**Figure 5:**
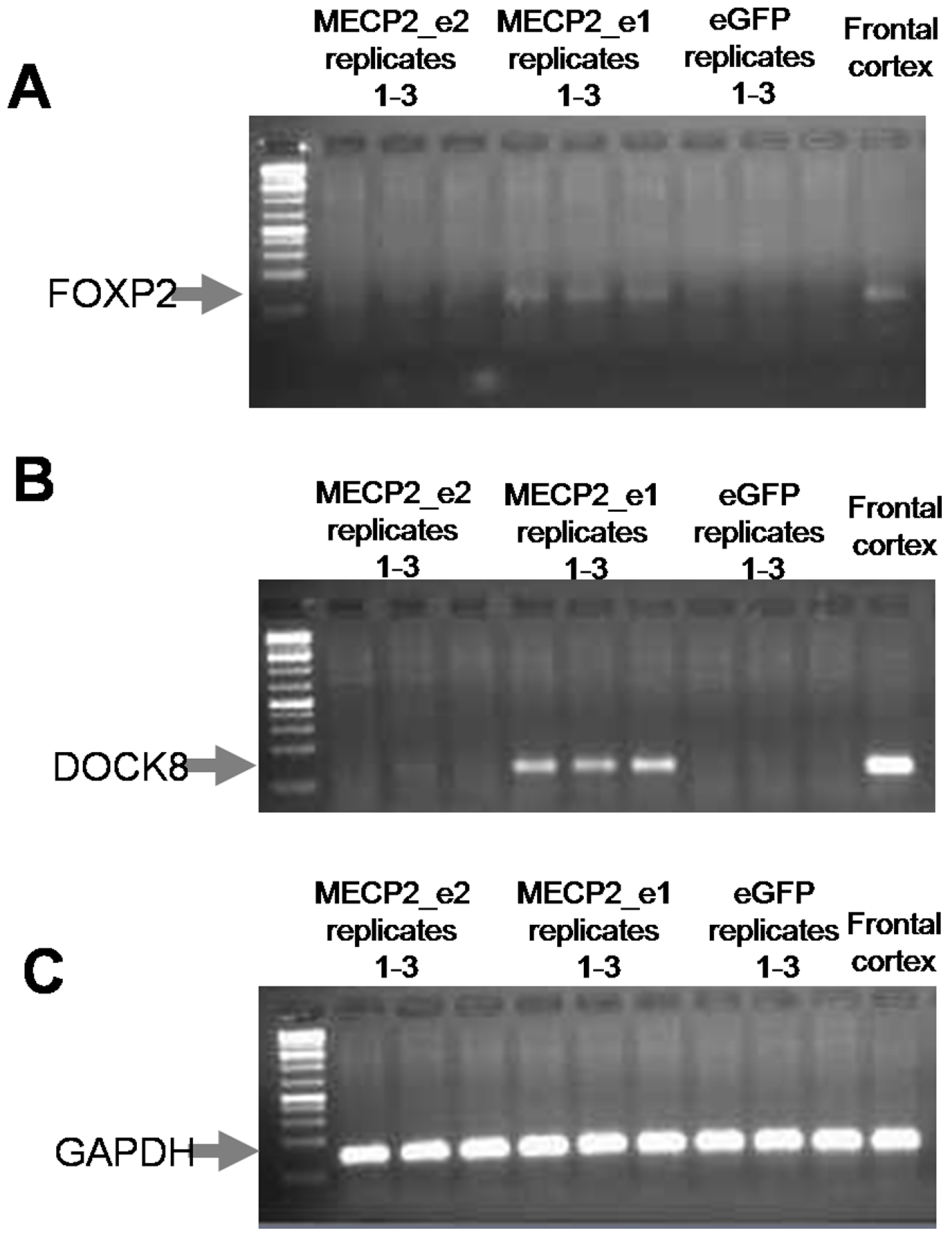
In SK-N-SH cells, MECP2_e1 induces expression of A) *FOXP2* and B) *DOCK8*. A weak signal is also visible when cells are infected with MECP2_e2 lentivirus vector. Frontal cortex was used as a positive control. Replicates represent biological replicates i.e. repeated infections. For *FOXP2*, real-time RT-PCR products were run on an agarose gel. For *DOCK8*, end point PCR was performed using KAPA2G Fast HotStartReadyMix (KapaBiosystems, KK5601. PCR consisted of 35 cycles, and the annealing temperature was 60°C. C) GAPDH was used as an endogenous control for RT-PCR.

We used quantitative RT-PCR to verify microarray results for a total of 41 genes, including 29 that, in addition to individually significant change in expression level (uncorrected for FDR), are known to be involved in cognitive function or neuronal development, or are highly expressed in CNS tissues, or are implicated in Rett syndrome, intellectual disability or autism (Tables 2, 3 and 4, Figures 4, and 5). An additional 12 genes were identified as differently expressed in both the transfected SK-N-SH cells and in the mouse knockout fibroblasts (Table 5). As uncorrected statistical tests were used to identify differentially expressed genes by microarray, a higher rate of false-positives and false-negatives was expected. We confirmed significant up-regulation of several genes resulting from over-expression of the e1 isoform in SK-N-SH cells, including *NAV3* (5-fold level increase), *FOXP2* (induced by MECP2_e1 over-expression, and weakly induced by MECP2_e2 (“induced”-indicating that no expression was detectable in control eGFP-infected cells, thus no fold-change calculation was possible), Figure 5a), *NPY1R* (six-fold increase), *SYN3* (two-fold increase), and *SEMA3D* (six-fold increase). Both MECP2_e1 and MECP2_e2 up-regulated *GABRA2* (18-fold and three-fold increase, respectively), *KCNA1* (seven-fold and three-fold increase, respectively) and *FOXG1* (six-fold and three-fold increase, respectively). Down-regulation of expression in the presence of e1 was seen with *UNC5C*(40% down-regulation) and *RPH3A* (30% down-regulation). Expression levels of four genes determined by the qPCR contradicted microarray results: *SPRX2*, *ITGA3* and *NDN* were reported as down-regulated by microarray analysis but found to be up-regulated by qPCR, and *CNTN4*, which was identified as up-regulated by microarray analysis, was indicated as down-regulated by qPCR (Table 2). Several of the genes, while potentially relevant to RTT, had very low levels of expression and could not be validated by this method, including *DLGAP2*, *CRH*, *GABRB, GRIN2A* and *PTCHD1* (Table 2).

**Table 2:** Comparison of microarray results and qPCR validation data for neuronally differentiated SK-N-SH cells infected with either MECP2e1 or e2.

	Treatment (Infection)	Differentially Regulated Gene	Microarrayp-value	Fold Change by Microarray	Fold Changeby RT-PCR	RT-PCRF test p value	Chahrour^1^Null/WT	Chahrour^1^Tg/WT
**Confirmed**	MECP2e1	NPY1R	0.014	5.2	6.2±2.9	0.00016094		
	MECP2e1	SYN3	0.002	4.2	2.0±0.6	0.00012736		
	MECP2e1	GABRA2	0.014	3.8	18.4±10.5	0.01086709	−0.270	0.440
	MECP2e2	GABRA2	–	NC	3.5±2.2	0.01086709		
	MECP2e1	KCNA1	0.028	3.8	7.3±2.0	0.03643621		
	MECP2e2	KCNA1	–	NC	3.3±3	0.03643621		
	MECP2e1	PTPRZ1	0.047	3.5	10.4±5.0	0.00359804		0.214
	MECP2e1	FOXG1B	0.007	3.7	5.7±7.3	0.23864889		
	MECP2e2	FOXG1B	0.019	2.8	2.7±2.7	0.23864889		
	MECP2e1	NAV3	0.008	6.5	5.3±1.0	0.00005067		
	MECP2e2	NAV3	0.046	5.5	0.7±0.2	0.00005067		
	MECP2e1	SEMA3D	0.032	3.0	5.8±5.1	0.09368591	−0.186	0.324
	MECP2e2	SEMA3D	0.030	2.3	0.6±0.5	0.09368591		
	MECP2e1	ELAVL3	0.034	0.2	0.8±0.2	0.185178		0.2
	MECP2e1	UNC5C	0.035	0.3	0.7±0.2	0.03063547		
	MECP2e1	RPH3A	0.012	0.3	0.7±0.3	0.02479987		
	MECP2e2	FMN2	0.021	0.3	0.9±0.3	0.03209883		
	MECP2e1	FOXP2	–	NC	Induced%	NA		
	MECP2e2	FOXP2	0.014	4.4	Induced%	NA		
**Inconclusive**	MECP2e1	CRH	0.016	0.1	*	NA	−2.06&	3.21&
	MECP2e1	GABRB1	6.863354E-4	7.4	*	NA		
	MECP2e1	PTCHD1	0.042	5.5	*	NA	−0.323	0.456
	MECP2e1	DLGAP2	0.005	5.4	*	NA	0.362	−0.306
	MECP2e1	GRIN2A	0.007	3.0	*	NA	1.47&	−1.88&
**Inconsistent or** **false calls**	MECP2e1	SRPX2^#$^	0.044	0.2	17.1±4.9	0.00030906	−0.334	0.192
	MECP2e1	NDN	0.028	0.3	3.3±1.2	0.00151035		
	MECP2e1	ITGA3	6.153462E-4	0.3	5.1±1.8	0.00039692	−0.282	0.319
	MECP2e1	CNTN4	0.005	5.9	0.5±0.3	0.32196181		
	MECP2e1	BMP7	0.039	3.3	0.8±0.4	0.05861708		0.302
	MECP2e1	CHL1	0.036	4.0	1.3±0.4	0.08370298		
	MECP2e2	REST	0.023	3.6	0.8±0.4	0.54730836		
	MECP2e1	NR0B1	1.20315E-4	0.1	8.3±3.2	0.00024781		
	MECP2e2	NR0B1	0.004	0.2	3.6±1.4	0.00024781		
	MECP2e1	NEFM	0.003	0.3	2.1±0.7	0.00429579		
	MECP2e2	NEFM	0.027	0.2	1.3±0.5	0.00429579		
	MECP2e1	GRM8	0.004	0.3	1.4±0.4	0.73088845		
	MECP2e2	GRM8	0.015	0.3	1.5±0.8	0.73088845		
	MECP2e1	LGICZ1	0.005	0.3	1.3±1.1	0.76438003		
	MECP2e2	LGICZ	0.006	0.3	1.2±0.5	0.76438003		

F test was calculated for pairwise comparison of RT-PCR results for MECP2e1 or e2 versus eGFP infection. Comparison with published data from knockout (null) and transgenic mouse gene expression microarray data (Chahrour et al, 2008 [33]) is shown. Overlap with several other gene expression/transcriptome analysis studies (Nectoux et al, 2010 [32]; Yakabe et al, 2008 [31]) is also commented on (see footnotes).

1 Chahrour et al, 2008.

% no expression detectable by qRT-PCR in control cells (eGFP infected), thus no fold-change calculation possible.

*too low to measure.

NA = not applicable (i.e. unable to calculate p-value).

NC = no call (i.e. microarray didn’t show any fold change in expression).

& from quantitative RT-PCR data.

# Also identified as up-regulated in transcriptome analysis of Rett patients; Nectoux et al, 2010 [32].

$ Also identified as up-regulated after siRNA knockdown of *MECP2* in human derived cell-lines; Yakabe et al, 2008 [31].

**Table 3:** Most highly induced or down-regulated genes in SK-N-SH cells over-expressing MECP2_e1 or MECP2_e2.

SK-N-SH Cells											
MECP2_e1 over-expression						MECP2_e2 over-expression					
Up-regulated			Down-regulated			Up-regulated			Down-regulated		
Gene	FC	qPCR	Gene	FC	qPCR	Gene	FC	qPCR	Gene	FC	qPCR
BCL11A	11.4	N/A	FLG	15.8	N/A	PSCD4	6.7	N/A	HS8ST3	5.4	N/A
PRLR	10.5	N/A	CCL2	13.9	N/A	BCL11A	6.0	N/A	NR0B1	5.0	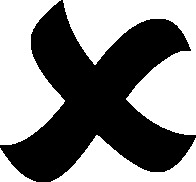
AMOT	10.3	N/A	CRH	11.5	?	PGK2	5.6	N/A	NEFM	4.6	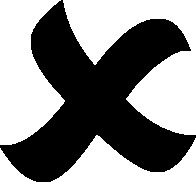
FGB	10.3	N/A	THBD	10.6	N/A	NAV3	5.5	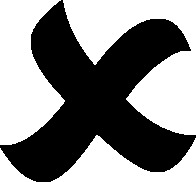	LRRN3	4.5	N/A
CTTNBP2	10.1	N/A	IL8	9.2	N/A	GJB2	5.4	N/A	LGICZ1	3.5	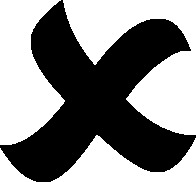
CD46	10.1	N/A	CRTC1	8.3	N/A	SIRPG	5.1	N/A	GRM8	3.4	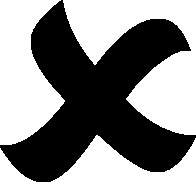
PTGS4	8.9	N/A	NR0B1	8.1	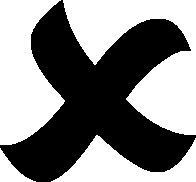	IGSF10	5.0	N/A	MAP2K6	3.4	N/A
PGK2	8.9	N/A	PLAU	6.5	N/A	DPEP1	5.0	N/A	MYOD1*	3.4	N/A
MYPN	8.7	N/A	CST2	6.4	N/A	KIR2DS4	4.8	N/A	SIX2	3.3	N/A
PLXNC1	8.5	N/A	IL1B	6.3	N/A	XAGE5	4.7	N/A	OTUD6A	3.3	N/A
PTPLAD2	8.5	N/A	ELAVL3	6.2	✓	SDPR	4.7	N/A	DHDH	3.2	N/A
FLVCR2	8.4	N/A	IVL	6.1	N/A	TBX1	4.6	N/A	PDK4	3.2	N/A
CFHR1	8.3	N/A	RGS33	6.0	N/A	CLEC4D	4.6	N/A	VMAC	3.1	N/A
DNAH11	8.2	N/A	NOV	6.0	N/A	CYP2E1	4.5	N/A	FMN2	3.1	✓
GRAP	8.1	N/A	SFTPA1	5.8	N/A	SLFN12	4.4	N/A	RHEB	3.0	N/A
ECM2	7.8	N/A	KLK6	5.8	N/A	FOXP2	4.4	✓	HIST1H2AC	3.0	N/A
LRIG3	7.8	N/A	OTOR	5.7	N/A	TUBB1	4.4	N/A	SYTL3	2.9	N/A
TMPRSS5	7.7	N/A	FAM84A	5.6	N/A	ARHGAP30	4.4	N/A	CYP4F2	2.9	N/A
FAM110C	7.6	N/A	PDGFC	5.6	N/A	ADH1A	4.4	N/A	ING1	2.9	N/A
LMX1B	7.5	N/A	EFEMP1	5.5	N/A	F2RL1	4.3	N/A	PSMD8	2.9	N/A

FC = fold change by microarray.

N/A = qPCR validation was not done.

✓ = qPCR results are in agreement with microarray results.

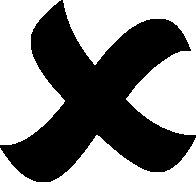
 = qPCR results contradict microarray results.

? = qPCR results were inconclusive.

* = similar change was observed in mouse Mecp2 knockout fibroblasts.

**Table 4:** Most highly induced or down-regulated genes in mouse Mecp2 knockout fibroblasts over-expressing MECP2_e1 or MECP2_e2.

Mouse Mecp2 knockout fibroblasts											
MECP2_e1 over-expression						MECP2_e2 over-expression					
Up-regulated			Down-regulated			Up-regulated			Down-regulated		
Gene	FC	qPCR	Gene	FC	qPCR	Gene	FC	qPCR	Gene	FC	qPCR
Fgg	7.2	N/A	Oxct2a	10.7	N/A	Stk38	3.1	N/A	Oxct2a	6.3	N/A
Pol3a	4.3	N/A	Podxl	8.9	N/A	Acss2	2.9	N/A	Hist1h1d	5.3	N/A
Slc7a6	4.3	N/A	Kcnmb2	8.1	N/A	Nkx6-2	2.9	N/A	Rfwd2	5.1	N/A
Slit2	4.1	N/A	Chrm1	7.2	N/A	Polb	2.9	N/A	Brf2	4.2	N/A
Ptprg	4.1	N/A	Pi16	7.2	N/A	Myocd	2.8	N/A	Cyp2c44	4.1	N/A
Slc35f1	4.0	N/A	Neurod2	7.0	N/A	Olfr557	2.7	N/A	Parp14	4.1	N/A
Ddef2	4.0	N/A	Kcnk1*	7.0	N/A	Evc	2.7	N/A	Rbm6	4.0	N/A
Dlg1	4.0	N/A	Rhbdl3	6.9	N/A	Fclr5	2.7	N/A	Rab31	4.0	N/A
Cul2	4.0	N/A	Fastkd1	6.9	N/A	Lce1g	2.7	N/A	Galm	3.8	N/A
Hmgn3	3.9	N/A	Cdh22*	6.7	N/A	Itga7	2.7	N/A	Olfr869	3.8	N/A
Slc1a3	3.9	N/A	Apoc4	6.5	N/A	Lce1a1	2.7	N/A	Cd209a	3.8	N/A
Rqcd1	3.9	N/A	Rad50	6.2	N/A	Oaz3	2.7	N/A	Sntg2	3.6	N/A
Nfkb1	3.8	N/A	Ccrl2	6.2	N/A	Igcf4	2.7	N/A	Brd8	3.6	N/A
Pkn2*	3.8	N/A	Prkcb1	6.0	N/A	Igf2bp3	2.7	N/A	Clec9a	3.6	N/A
Peli1	3.8	N/A	Olfr799	5.9	N/A	Rab6b	2.6	N/A	Grin2d	3.5	N/A
Plcxd2	3.8	N/A	Cd4	5.8	N/A	Ifng	2.6	N/A	Slc22a6	3.4	N/A
Bcl7b	3.7	N/A	Gabrd	5.8	N/A	Olfm2	2.6	N/A	Smarce1	3.3	N/A
Shoc2	3.7	N/A	Pde1c	5.8	N/A	Atoh7	2.6	N/A	Kif14*	3.3	N/A
Uap1l1	3.7	N/A	Olfr869	5.8	N/A	Enpep	2.6	N/A	Mapk9	3.3	N/A
Adam30	3.7	N/A	Serpinb12	5.7	N/A	Cdc14b*	2.6	N/A	Hemt1	3.1	N/A

FC = fold change by microarray.

N/A = qPCR validation was not done.

* = similar change was observed in SK-N-SH cells.

**Table 5:** Genes identified as differentially expressed in both the transfected human (SK-N-SH) and mouse (Mecp2 knock-out fibroblast) cells.

	Treatment (Infection)	Different-ially Regulated Gene	Fold Change in SK-N-SH Cells by Microarray	Fold Change in Primary Mouse Fibroblasts by Microarray	Fold Change in SK-N-SH Cells by Real-Time RT-PCR	Fold Change in Primary Mouse Fibroblasts by Real-Time RT-PCR	Chahrour^1^ Null/WT	Chahrour^1^ Tg/WT
**Confirmed**	MECP2e1	DOCK8	3.1 (p = 0.004)	2.0 (p = 0.015)	Induced by e1%	1.3±0.5 (p = 0.269)		
**Inconclusive**	MECP2e1	TSHR	4.1 (p = 0.042)	2.3 (p = 0.016)	*	*		
	MECP2e1	BMX	3.7 (p = 0.049)	2.9 (p = 0.044)	3.4±0.9 (p = 0.333)	1.5±0.5 (p = 0.226)		
	MECP2e1	KCNJ9	0.4 (p = 0.014)	0.5 (p = 0.009)	*	*		
	MECP2e1	ZBTB10	0.5 (p = 0.005)	0.5 (p = 0.014)	0.9±0.6 (p = 0.811)	*		
	MECP2e1	RNF2	2.6 (p = 0.004)	2.3 (p = 0.013)	1.9±0.9 (p = 0.376)	2.2±0.2 (p = 0.777)	−0.131	0.387
	MECP2e1	COQ10B	2.9 (p = 0.035)	2.4 (p = 0.038)	1.2±0.1 (p = 0.428)	1.7±0.5 (p = 0.560)		
	MECP2e1	LPHN3	2.4 (p = 0.022)	2.1 (0.015)	3.5±1.3 (p = 0.946)	1.9±1.7 (p = 0.362)		
**Inconsistent or false calls**	MECP2e1	ITGBL1	0.4 (p = 0.015)	0.3 (p = 0.021)	3.7±0.1 (p = 1.62E-06)	5.0±1.3 (p = 0.197)		
	MECP2e1	GPR85	0.4 (p = 0.009)	0.3 (p = 0.015)	1.4±0.2 (p = 0.008)	3.7±1.8 (p = 0.199)	0.396	−0.250
	MECP2e2	TMC4	2.3 (p = 0.028)	2.1 (p = 0.005)	0.7±0.6 (p = 0.028)	1.4±1.0 (p = 0.490)	0.414	−0.157
	MECP2e2	SMCR8	0.4 (p = 0.008)	0.4 (p = 0.048)	1.0±0.3 (p = 0.533)	2.1±1.2 (p = 0.552)		

Comparison with published data from knockout (null) and transgenic mouse gene expression microarray data (Chahrour et al, 2008 [33]) is also shown.

1 Chahrour et al, 2008 [33].

% no expression detectable by qRT-PCR in control cells (eGFP infected), thus no fold-change calculation possible.

*too low to measure.

Of the twelve genes identified to be differentially regulated by MECP2 isoforms in both human and mouse cells, we were able to confirm induction of *DOCK8* expression by MECP2_e1 in neuronally differentiated SK-N-SH cells (Figure 5b). A slight increase in *Dock8* expression in primary mouse MeCP2 knockout fibroblasts infected with MECP2_e1 lentivirus vector was not statistically significant. Five genes (*BMX, ZBTB10*, *RNF2*, *COQ10B* and *LPHN3*) showed expression trends by qRT-PCR, in accordance with microarray results, in either human or mouse cells, or both, but the expression level changes were not statistically significant. Two of the genes, *TSHR* and *KCNJ9*, had very low expression levels that could not be quantified. Four genes (*ITGBL1*, *GPR85*, *TMC4* and *SMCR8*) showed expression level changes, by quantitative RT-PCR, that contradicted microarray findings. Results of microarray and quantitative RT-PCR for genes differentially regulated by MECP2 isoforms in both human and mouse cells are summarized in Table 5.

## Discussion

We have used an over-expression system to identify downstream targets of both the main isoforms of MECP2. This allows us some insight into the differences and similarities between the two isoforms. The use of an over-expression system obviously does not mimic the situation in Rett patients, and, as mentioned earlier, is more etiologically relevant to the MECP2 duplication syndrome. However, the purpose of this approach was chiefly to produce a strong and measurable effect from the addition of the exogenous MECP2 isoforms. Another caveat is that direct comparison of levels of up- or down-regulation of genes for the two isoforms would be inappropriate, as the translational efficiencies of the two isoforms are different [3], and stability and longevity are also predicted to be different (see *in silico* analysis in Introduction). Another restriction of this approach is that it cannot distinguish whether MECP2 is a direct regulator, or whether it is acting through other effector molecules. Chromatin-immunoprecipitation followed by sequencing (ChIP-seq) may help show which of these genes are direct targets, however, recent studies using this approach suggest that MECP2 does not show discreet binding targets, but rather shows a much broader genome-wide distribution that generally tracks the density of CpG methylation [16]. It is thus likely that the gene-specificity for MECP2 depends very much upon the presence of co-repressors or activators, which may in turn be tissue and temporally specific.

Our quantification of e1 and e2 mRNA in eGFP-infected SK-N-SH cells and *post mortem* human frontal cortex using the ΔΔCt method of relative quantification shows ∼9-fold higher levels of e2 than e1. This contrasts with several previous studies which used quantification of end-point RT-PCR products and showed higher levels of e1 than e2 [2], [3]. Our previous study looking at e1 and e2 mRNA levels in lymphocytes using the ΔΔCt method also showed very similar levels of e1 and e2 [17], also differing from the previous reports [2], [3]. This may not, however, correspond directly to relative amount of the protein isoforms, as e1 is translated with much higher efficiency than e2 [3], and has been shown to be a more stable protein than e2 [10], and as predicted by in silico analysis of the different N-terminal sequences for likelihood of N-methionine excision and N-terminal acetylation and effect on protein stability and longevity [10].

Of the isoform-specific targets that were validated using qPCR, *NAV3* is one of the most interesting, and showed approximately five-fold increase in expression with over-expression of MECP2_e1. *NAV3* encodes neuron navigator 3- so called based on sequence similarity to the C-terminal end of the *C. elegans* protein unc-53, which is involved in axon guidance. Affymetrix gene chip data shows *NAV3* to be strongly expressed in brain, and in particular fetal brain, prefrontal cortex and amygdala (microarray data for probe 204823_AT from GeneAtlas U133A; biogps.org). Its expression correlates with that of *MEF2C* (probe 209200_AT)- a gene linked with severe intellectual disability (ID), stereotypic movements and epilepsy [18]. Zweier et al. [19] reported several patients with *de novo* truncating or missense mutations in *MEF2C*, and showed decreased levels of *MECP2* and *CDKL5* expression in patient lymphocytes, and speculated that the phenotypic overlap between RTT and *MEF2C* mutation patients is due to a common pathway.

*SEMA3D* showed a six-fold increase in expression with MECP2_e1. *SEMA3D* encodes a semaphorin protein. Semaphorins are secreted molecules that function primarily through neuropilin receptors to guide the axonal growth cone. Liu and Halloran [20]showed that SEMA3D protein expression at the dorsal spinal cord midline in zebrafish repelled growth of peripheral axons from the spinal cord, and knockdown reduced numbers of peripheral axons. SEMA3D repels axons through receptors containing neuropilin-1A, and attracts axons through receptors containing neuropilin-1A and 2B [21].

*NPY1R* expression was also increased approximately six-fold under MECP2_e1. *NPY1R* encodes the neuropeptide Y receptor 1. Neuropeptide Y, or NPY, is a 36-amino-acid neurotransmitter, involved in neurophysiological functions such as cardiovascular homeostasis [22] and hypothalamic regulation of food intake [23]. NPY has several receptors, including NPY1R [24]. *NPY1R* also shows strong expression almost exclusively in fetal brain (microarray data for probe 205440_s_at from GeneAtlas U133A; biogps.org). Interestingly, Rett syndrome includes dysregulation of cardiovascular functioning, described as “an increase in sympathetic activity that is not counter balanced by adequate vagal tone” [25]. It should also be noted that the isoform-specific localization for MECP2 in mouse brains, found isoform e1 but not e2 in the hypothalamus [6].

The gene *SYN3*, which encodes Synapsin 3, a vesicle-associated phosphoprotein, located at the synapse, is also significantly over-expressed (∼two-fold) in SK-N-SH cells under regulation of MECP2_e1. Its expression is strongly correlated with that of KCNH6- a potassium voltage-gated channel, subfamily H (microarray data for probe 206322_at (SYN3) and 211045_s_at (KCNH6) from GeneAtlas U133A; biogps.org).

We validated two genes that were down-regulated with the over-expression of MEPC2_e1. These were *UNC5C* (decreased by 40%) and *RPH3A* (decreased by 30%). *UNC5C* belongs to a family of netrin-1 (MIM 601614) receptor genes and is thought to mediate the chemorepulsive effect of netrin-1 on specific axons. *RPH3A* (MIM *612159) encodes Rabphilin 3A, an effector of RAB3A (MIM 179390), a G-protein known to be involved in exocytosis during synaptic neurotransmission, interacting with the SNARE synaptic complex [26], [27]. *RPH3A* is strongly expressed in whole brain, prefrontal cortex, cingulate cortex, subthalamic nucleus, and thalamus in particular (microarray data for probe 205230_AT from GeneAtlas U133A; biogps.org).

Up-regulation of *FOXP2* (MIM*605317) by MECP2 (both isoforms) is of significance because of the association between this gene and severe speech and language disorder known as developmental verbal dyspraxia (SPCH1; MIM 602081; [28]). Partial or complete loss of acquired spoken language is one of the main diagnostic criteria for Rett syndrome [29], however SPCH1 is associated with articulatory disturbance and severe orofacial dyspraxia [30] – a very different clinical manifestation. Microarray analyses on COS-7 cells transfected with a plasmid (non-lentiviral) MECP2_e1 construct also showed a >2-fold up-regulation of *FOXP2* (data not shown). One of the genes showing down-regulation under MECP2_e1 by microarray, *SRPX2*, actually showed significant up-regulation by RT-PCR analysis (17-fold; p = 3×10^−4^). This gene, which encodes a sushi repeat-containing protein, was also inferred as down-regulated by *MECP2* in microarray analysis in several studies of human cell lines [31], [32], but up-regulated in the study of hypothalami from null mice [33]. Disruption of *SRPX2* is associated with Rolandic epilepsy, speech dyspraxia and ID [34]. It has also been shown that the SRPX2/uPAR complex is regulated by FOXP2, thus suggesting SRPX2 may play a role in speech-related disorders [35]. It is possible that the down-regulation of *SRPX2* seen in our study is mediated by the up-regulation we see for *FOXP2*, and that this together may play a significant role in the phenotypic features of RTT.

Both isoforms appear to up-regulate expression of *GABRA2*, a gamma-aminobutyric acid A receptor gene located on chromosome 4p. The GABA receptor gene cluster at this locus has previously been implicated in autism [36]–[38], and GABA(A) receptors including GABRA2 have been reported to be significantly down-regulated in brains of autism subjects [39]. Dysfunction of GABA signaling is believed to underlie various Rett syndrome-like phenotypes seen in Mecp2-deficient mice, in particular forepaw stereotypic movements, increased sociability, impaired motor coordination, cognitive deficits, and respiratory dysrhythmia [40]. Our data suggests that disruption of either MECP2 isoform, would down-regulate *GABRA2* expression.*KCNA1* is also upregulated by both MECP2 isoforms. *KCNA1* encodes a voltage-gated potassium channel, which binds to postsynaptic density proteins SAP97, PSD-93 and PSD-95. Mutations in *KCNA1* may result in seizures, episodic ataxia and myokymia syndrome (MIM 160120).

Mutations in *FOXG1* have recently been shown to be the cause of a syndrome frequently referred to as a congenital form of Rett (MIM 613454), typically with *de novo* heterozygous truncating mutations associated with infantile onset of microcephaly, ID, no speech development, poor motor development and stereotypic movements [41]–[44]. Interestingly, it has recently been shown that MECP2_e2, but not e1, promotes neuronal death, and that this activity is limited through interaction with FOXG1. Thus, it is of great interest that *FOXG1* should be validated as a downstream target of MECP2, and for both isoforms (MECP2_e1: six-fold increase; MECP2_e2: three-fold increase). Both isoforms of MECP2 have recently been shown to interact directly with the FOXG1 gene, with higher level of interaction of MECP2_e2 than for _e1 [45].

A number of other genes of interest were identified through analysis of the microarray data as being over- or under-expressed, but because expression levels were at the low end in SK-N-SH cells, it was impossible to validate the findings with any reliability. These genes included several previously identified in relation to Rett, autism or ID, such as *CRH* (MIM*122560; Corticotropin-releasing hormone; dysregulated *CRH* expression shown in Mecp2 knockout mice; [33], [46]), *PTCHD1* (MIM*300828; disruptions may result in autism or ID; [47], [48]), *GRIN2A* (MIM*138253; a component of synaptic glutamate receptors; mutations have been associated with seizures and ID, [49]), *DLGAP2* (MIM*605438; CNVs disrupting this gene have been identified in autism; [50]).

The up-regulation of genes such as *DOCK8*, confirmed by qRT-PCR, in both SK-N-SH cells and primary mouse MeCP2 knockout fibroblast is also of much interest. Defects in *DOCK8* have been reported as the cause of autosomal dominant ID type 2 (MRD2; MIM 614113 [51]). However, somewhat in contradiction to this, recessive mutations in this gene have been reported as the cause of Hyper IgE recurrent infection syndrome (MIM 243700 [52]), for which there is no cognitive deficit reported. Down-regulation of genes such as *SMCR8* was observed in both SK-N-SH cells and Mecp2-null mouse fibroblasts. *SMCR8* is a candidate gene for Smith–Magenis Syndrome (SMS), a developmental disorder that affects many parts of the body [53]. The major features of this condition include ID, facial features, sleep disturbances, and behavioral problems.

The identification and validation of various synapse-related genes showing altered transcription after *MECP2* over-expression supports the hypothesis that mutations in *MECP2* cause Rett syndrome through disruption of synaptic plasticity [54]. The results indicating that both isoforms up-regulate some of the same genes, e.g. *KCNA1*, *FOXG1*, and *GABRA2*, suggests that genetic disruption of the *MECP2_e2* isoform could potentially have a phenotypic effect, however one would expect it to be milder than for disruption involving *MECP2_e1*, given the differences in location of the two isoforms in the brain [6]. Differences in the gene targets between the two isoforms may reflect actual differences in gene targets, however it cannot be ruled out that some of the differences may be downstream effects of the over-expression, or may be due in part to differences in protein stability and longevity within the infected cells. The fact that some qPCR results contradict the microarray data suggests that there are limitations to the conclusions that may be drawn on unvalidated microarray gene expression data. Moves towards transcriptome analysis using next generation sequencing approaches should improve the accuracy for determining gene targets for MECP2.

The gene ontology analysis reported here seems to show a more diverse set of functions for e1, compared to e2, and in particular an effect of e1 on regulation of genes involved in synaptic transmission, axonogenesis and activity of voltage-gated channels (SK-N-SH cells), and neuronal differentiation and development (fibroblasts). In contrast, many genes up- or down-regulated by e2 appear to be involved in chromatin organization and transcriptional regulation. In both SK-N-SH and fibroblasts, the genes down-regulated by both isoforms are categorized as being involved in transcriptional regulation.

Differences at the N-termini of the two MECP2 isoforms are predicted to generate an N-terminal N-acetylated alanine for MeCP2_e1, but a non-acetylated valine for MeCP2_e2. This is likely to result in a much shorter lifespan for the e2 isoform [8]. Whether this alone is enough to influence the gene regulatory effects of MeCP2, or whether it may also have a direct effect on DNA-binding specificity, remains to be seen.

In summary, we provide evidence that the two isoforms of MECP2 show distinct but overlapping profiles of gene transcription under their regulation, and propose that a number of these transcriptional effects translate to the clinical features of Rett syndrome.

## Supporting Information

Figure S1 **Infection efficiency determined by eGFP expression in recombinant lentivirus treated cells.** A) DAPI was used to visualize nuclei and determine the total number of cells in the visual field. Infection efficiency was calculated as the percentage of eGFP positive cells. SK-N-SH cells are shown. B) Comparison of infection efficiency between SK-N-SH cells and primary mouse Mecp2 knockout fibroblasts. MOI used is indicated above the bars. Calculation was done based on three independent infections for each sample.
(TIF)

Figure S2 **Neuronal differentiation of neuroblastoma cell line SK-N-SH.** A) Untreated cells. B) Cells treated with retinoic acid only. C) Cells treated with retinoic acid and herbimycin A.
(TIF)

Figure S3 **Induction of MECP2 expression in mouse Mecp2 knockout fibroblast by stable infection with recombinant lentiviruses expressing MECP2 isoforms e1 and e2.** Real-time RT-PCR amplification curves for MECP2_e1 (first row), MECP2_e2 (second row) and endogenous control Tbp (third row) are shown. Mecp2 knockout mouse fibroblasts were stably infected by recombinant lentiviruses encoding either MECP2_e1 (first column), MECP2_e2 (second column) or eGFP control (third column).
(TIF)

Table S1 **Uncorrected one way ANOVA (p<0.05) results of gene expression microarray on neuronally differentiated SK-N-SH cells infected with MECP2_e1, MECP2_e2 or eGFP lentiviral vectors.**
(XLSX)

Table S2 **Uncorrected one way ANOVA (p<0.05) results of gene expression microarray on mouse Mecp2 knockout fibroblasts infected with MECP2_e1, MECP2_e2 or eGFP lentiviral vectors.**
(XLSX)

Table S3 **Gene expression microarray results for differentiated SK-N-SH cells with over-expression of MECP2_e1 compared to cells infected with eGFP (uncorrected t-test).**
(XLS)

Table S4 **Gene expression microarray results for differentiated SK-N-SH cells with over-expression of MECP2_e2 compared to cells infected with eGFP (uncorrected t-test).**
(XLS)

Table S5 **List of genes showing altered transcription in differentiated SK-N-SH cells under e1 or e2 over-expression (or both), categorized as either down-regulation or up-regulation.** Overlap with previous transcriptome studies for MECP2 is also indicated, with genes highlighted in yellow reported as up-regulated or activated, and in green as down-regulated or repressed.
(XLSX)

Table S6 **Gene expression microarray results for mouse Mecp2 knockout fibroblasts with over-expression of MECP2_e1 compared to cells infected with eGFP (uncorrected t-test).**
(XLS)

Table S7 **Gene expression microarray results for mouse Mecp2 knockout fibroblasts with over-expression of MECP2_e2 compared to cells infected with eGFP (uncorrected t-test).**
(XLS)

Table S8 **List of genes showing altered transcription in mouse Mecp2 knockout fibroblasts under e1 or e2 over-expression (or both), categorized as either down-regulation or up-regulation.**
(XLSX)

SKNSH S1 **DAVID Gene Ontology analysis (** http://david.abcc.ncifcrf.gov/ **[14], [15])** for functional clustering of genes down regulated in SKNSH cells by both e1 and e2.
(TXT)

SKNSH S2 **DAVID Gene Ontology analysis (** http://david.abcc.ncifcrf.gov/ **[14], [15])** for functional clustering of genes down regulated in SKNSH cells by e1.
(TXT)

SKNSH S3 **DAVID Gene Ontology analysis (** http://david.abcc.ncifcrf.gov/ **[14], [15]** **) for functional clustering of genes down regulated in SKNSH cells by e2.**
(TXT)

SKNSH S4 **DAVID Gene Ontology analysis (** http://david.abcc.ncifcrf.gov/ **[14], [15])** for functional clustering of genes upregulated in SKNSH cells by both e1 and e2.
(TXT)

SKNSH S5 **DAVID Gene Ontology analysis (** http://david.abcc.ncifcrf.gov/ **[14], [15]) for functional clustering of genes upregulated in SKNSH cells by e1.**
(TXT)

SKNSH S6 **DAVID Gene Ontology analysis (** http://david.abcc.ncifcrf.gov/ **[14], [15])** for functional clustering of genes upregulated in SKNSH cells by e2.
(TXT)

Mouse S1 **DAVID Gene Ontology analysis (** http://david.abcc.ncifcrf.gov/ **[14], [15]**)** for functional clustering of genes down regulated in mouse knockout fibroblasts by e1.**
(TXT)

Mouse S2 **DAVID Gene Ontology analysis (** http://david.abcc.ncifcrf.gov/ **[14], [15])** for functional clustering of genes down regulated in mouse knockout fibroblasts by e2.
(TXT)

Mouse S3 **DAVID Gene Ontology analysis (** http://david.abcc.ncifcrf.gov/ **[14], [15])** for functional clustering of genes down regulated in mouse knockout fibroblasts by both e1 and e2.
(TXT)

Mouse S4 **DAVID Gene Ontology analysis (** http://david.abcc.ncifcrf.gov/ **[14], [15])** for functional clustering of genes upregulated in mouse knockout fibroblasts by e1.
(TXT)

Mouse S5 **DAVID Gene Ontology analysis (** http://david.abcc.ncifcrf.gov/ **[14], [15])** for functional clustering of genes upregulated in mouse knockout fibroblasts by e2.
(TXT)

Mouse S6 **DAVID Gene Ontology analysis (** http://david.abcc.ncifcrf.gov/ **[14], [15])** for functional clustering of genes upregulated in mouse knockout fibroblasts by both e1 and e2.
(TXT)
