# Supplementary figures and images for: Over-Expression of Either MECP2_e1 or MECP2_e2 in Neuronally Differentiated Cells Results in Different Patterns of Gene Expression
Source: PLoS One. 2014 Apr 3;9(4):e91742. doi: 10.1371/journal.pone.0091742 (PMC3974668; doi:10.1371/journal.pone.0091742)

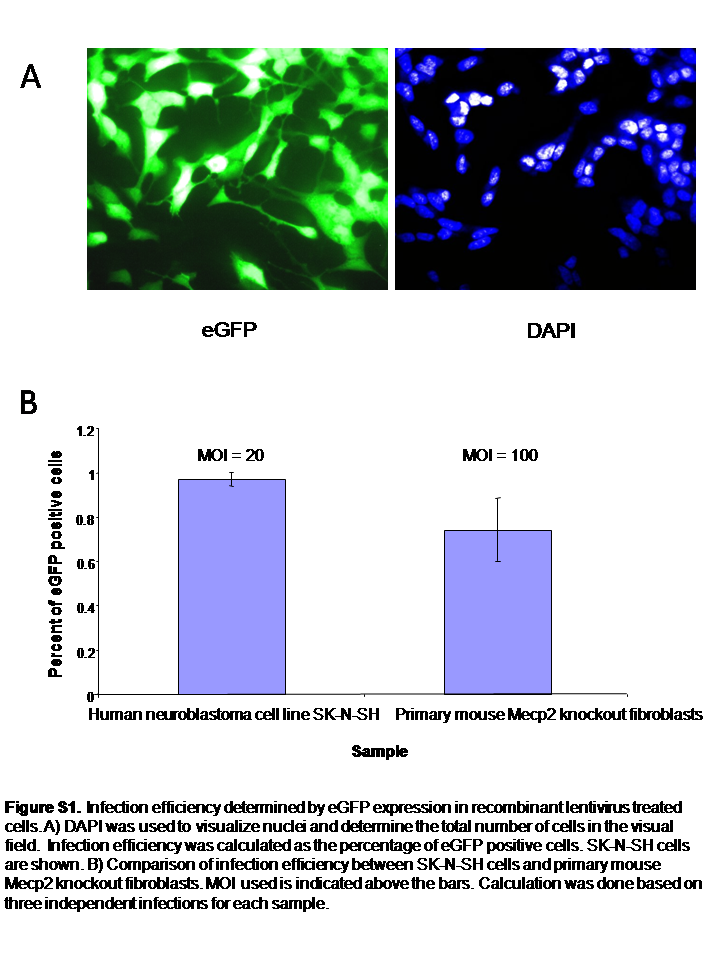

Supplement: Figure S1 — Infection efficiency determined by eGFP expression in recombinant lentivirus treated cells. A) DAPI was used to visualize nuclei and determine the total number of cells in the visual field. Infection efficiency was calculated as the percentage of eGFP positive cells. SK-N-SH cells are shown. B) Comparison of infection efficiency between SK-N-SH cells and primary mouse Mecp2 knockout fibroblasts. MOI used is indicated above the bars. Calculation was done based on three independent infections for each sample. (TIF) [file pone.0091742.s001.tif]

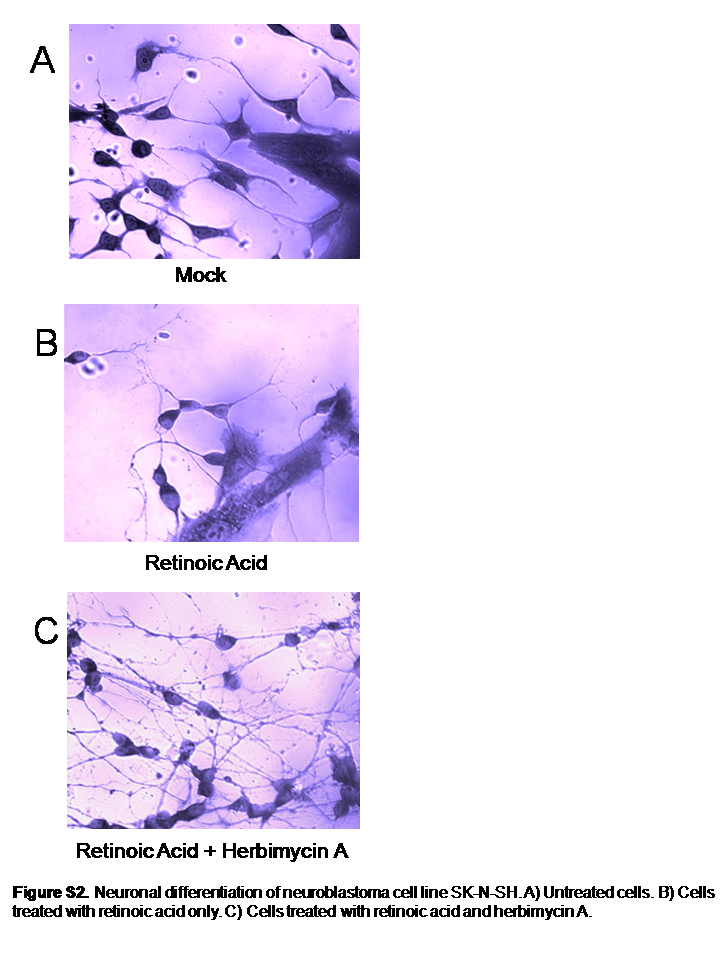

Supplement: Figure S2 — Neuronal differentiation of neuroblastoma cell line SK-N-SH. A) Untreated cells. B) Cells treated with retinoic acid only. C) Cells treated with retinoic acid and herbimycin A. (TIF) [file pone.0091742.s002.tif]

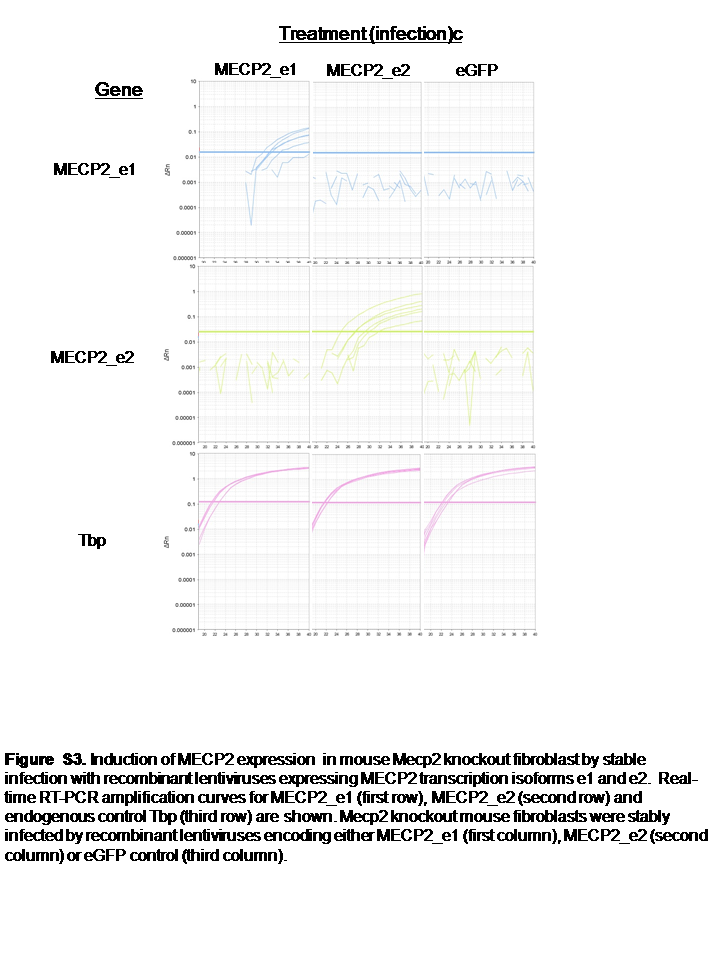

Supplement: Figure S3 — Induction of MECP2 expression in mouse Mecp2 knockout fibroblast by stable infection with recombinant lentiviruses expressing MECP2 isoforms e1 and e2. Real-time RT-PCR amplification curves for MECP2_e1 (first row), MECP2_e2 (second row) and endogenous control Tbp (third row) are shown. Mecp2 knockout mouse fibroblasts were stably infected by recombinant lentiviruses encoding either MECP2_e1 (first column), MECP2_e2 (second column) or eGFP control (third column). (TIF) [file pone.0091742.s003.tif]
